# Genetic health and population monitoring of two small black bear (*Ursus americanus*) populations in Alabama, with a regional perspective of genetic diversity and exchange

**DOI:** 10.1371/journal.pone.0186701

**Published:** 2017-11-08

**Authors:** John P. Draper, Lisette P. Waits, Jennifer R. Adams, Christopher L. Seals, Todd D. Steury

**Affiliations:** 1 School of Forestry and Wildlife Sciences, Auburn University, Auburn, Alabama, United States of America; 2 Department of Fish and Wildlife Sciences, University of Idaho, Moscow, Idaho, United States of America; National Cheng Kung University, TAIWAN

## Abstract

One of the major concerns in conservation today is the loss of genetic diversity which is a frequent consequence of population isolation and small population sizes. Fragmentation of populations and persecution of carnivores has posed a substantial threat to the persistence of free ranging carnivores in North America since the arrival of European settlers. Black bears have seen significant reductions in range size from their historic extent, which is most pronounced in the southeastern United States and even more starkly in Alabama where until recently bears were reduced to a single geographically isolated population in the Mobile River Basin. Recently a second population has naturally re-established itself in northeastern Alabama. We sought to determine size, genetic diversity and genetic connectivity for these two populations in relation to other regional populations. Both populations of black bears in Alabama had small population sizes and had moderate to low genetic diversity, but showed different levels of connectivity to surrounding populations of bears. The Mobile River Basin population had a small population size at only 86 individuals (76–124, 95% C.I.), the lowest genetic diversity of compared populations (richness = 2.33, Ho and He = 0.33), and showed near complete genetic isolation from surrounding populations across multiple tests. The newly recolonizing population in northeastern Alabama had a small but growing population doubling in 3 years (34 individuals 26–43, 95% C.I.), relatively moderate genetic diversity compared to surrounding populations (richness = 3.32, Ho = 0.53, He = 0.65), and showed a high level of genetic connectivity with surrounding populations.

## Introduction

One of the major concerns in conservation biology is the loss of genetic diversity [1], which is a frequent consequence of population isolation and small population sizes [2]. Small, isolated populations are at greater risk for loss of genetic diversity due to increased potential for genetic drift and inbreeding [3]. As genetic diversity is lost, fitness and fecundity of individuals in a population can be reduced, resulting in inbreeding depression [4]. Inbreeding depression is the first step in genetic meltdown, the fixation of deleterious mutations [5], which in turn leads to a negative feedback loop of continued reduction of both population size and genetic diversity. Such feedback loops are known as an extinction vortex, and can ultimately lead to the loss of a population [6]. The population wide effect of a loss of diversity combined with its self-accelerating nature can make a low or decreasing level of genetic diversity of particular concern.

Various factors such as trophic level, home range size, and reproduction rate make certain taxonomic groups of species at higher risk for these adverse population and genetic outcomes [7]. For example, populations of animals from within the order *Carnivora* are especially prone to population isolation, reduced population size, and ultimately loss of genetic diversity due to greater persecution, large home ranges, and high trophic level [8]. Persecution of carnivores arises from actual and perceived threats to human interests and safety, and can contribute to populations being reduced [9]. Furthermore, carnivores large home ranges, increases their exposure to anthropogenic persecution and utilization [10]and vulnerability to habitat fragmentation and consequently population isolation within protected areas or other refugia [7,10,11]. Finally, carnivores’ high trophic level, combined with their typically low recruitment rate and naturally low densities, further contribute to their tendency towards isolated populations with few individuals [7]. Once reduced to small, isolated populations, carnivores are especially vulnerable to genetic meltdown, and local extinction of a population. Thus, wildlife managers need to estimate and monitor population sizes, genetic connectivity between populations and genetic diversity of carnivore populations to ensure their long-term survival.

Black bears (*Ursus americanus*) have suffered particularly sharp declines in population size and range in North America, creating conditions that would increase population isolation and decrease effective population sizes. Black bear ranges have been reduced 55% from the historical extent in North America, with an even more significant 80% reduction in the southeastern United States [12–14]. As a result, black bears in Louisiana, for example, have shown extremely low genetic diversity and significant genetic population structure indicating a restriction in gene flow between populations [15]. Studies of other isolated bear populations in the Southeast are needed to identify and prevent similar situations.

Alabama has two small populations of black bears, one of which is geographically isolated from other populations of bears. This population, which was restricted to the lowlands surrounding the Mobile River Basin (MRB; [16]), has persisted through the extensive habitat conversion and persecution that accompanied European settlement of the area. However, the U.S. Fish and Wildlife Service suggested that the MRB population had a low probability for long-term persistence due to its small size and isolation from other bear populations [17]. Furthermore, Kasbohm et al. (1994 [18], as reported in Edwards 2002[19]) found potential physical expression of low genetic diversity, including cryptorchidism, prolapsed rectum, and kinked or absent coccygeal vertebrae. The second population of black bears in Alabama is a newly recolonizing population in northeastern Alabama (NAL), whose source population and level of continuing connectivity with its source is unknown. Little is known about either populations’ current status; the MRB population was last studied over 15 years ago [19] and the NAL population has never been studied. Therefore, wildlife managers need to better understand the current genetic status and potential for continued genetic health for both populations.

In this study we aimed to evaluate the genetic diversity of these two populations and measure the factors that can influence diversity including population size and genetic connectivity to surrounding populations. Our objectives were to: 1. estimate the abundance and distribution of black bears in the core of both the MRB and NAL populations; 2. evaluate genetic connectivity with surrounding populations and 3. estimate the genetic diversity within each population and compare it to the genetic diversity of surrounding populations of black bears in the Southeastern United states. Such information will be useful for guiding future management actions aimed at promoting genetic diversity and in turn the fitness of black bears in Alabama.

## Methods

### Ethics statement

All sampling was done in accordance with the recommendations and permitting of the Auburn University Institutional Animal Care and Use Committee (protocol number 2012–2197) who approved this study. All samples were collected from properties with the permission of the private land owners or relevant land management agency. No permit was required from the state of Alabama, for non-invasive sampling.

### Study area

**Fig 1:**
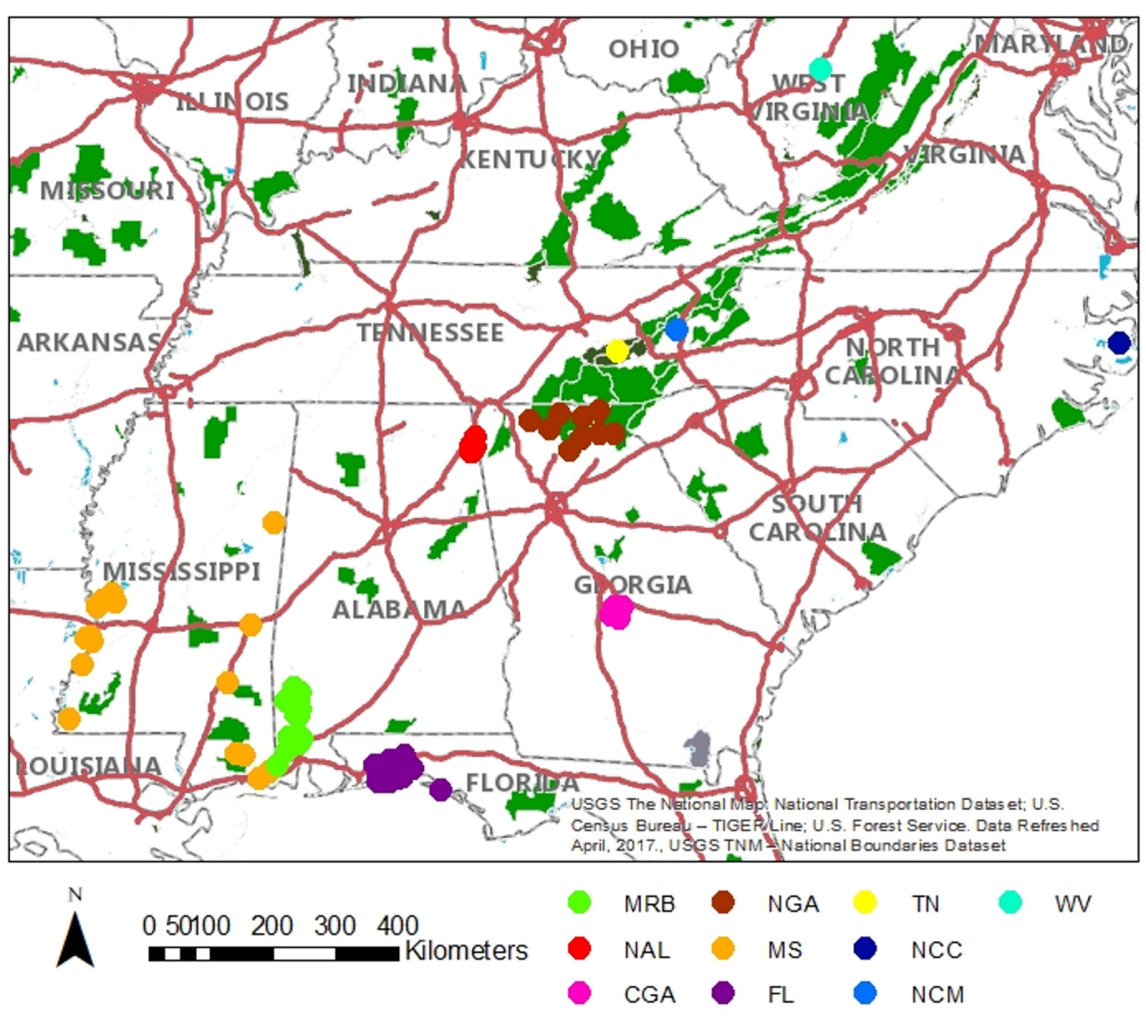
Sample collection locations. GPS locations of samples collected from MRB, NAL, MS, CGA, FL, centers of county collected for NGA and approximate center of study area for TN, NCC, NCM, and WV where location information was not available for individual samples.

Bears within Alabama were sampled from two breeding populations. The MRB population samples were collected from Mobile and Washington counties in the south of the state between the Mississippi border and the Tombigbee River. The NAL samples were collected in the northeastern corner of the state in a small portion of Cherokee and DeKalb counties in and around Little River Canyon National Preserve. These two areas represent the entirety of breeding populations of black bears in Alabama (Fig 1).

Within the MRB study area, bears utilized both natural and human-dominated landscapes. Natural and near natural habitat available to bears in the MRB varied from woody wetlands to pine plantations [20]. Bears also utilized areas in close proximity to suburban and exurban homes, often frequenting yards without the knowledge of the homeowners. Roughly 500,000 people lived within the sampling area at the time of study [21].

The NAL bear population had a core distribution in Little River National Preserve (LIRI) in DeKalb County. The habitat in this region was mountainous and dominated by deciduous forests and pine plantations [20]. In addition to federally managed lands including LIRI and Talladega National Forest, many large tracts of land were managed privately for hunting and/or timber production, providing much more continuous habitat than seen in the MRB. Similarly, human population density was lower, with only roughly 130,000 people residing within parts of the study area potentially occupied by bears [21].

Five comparison populations outside of Alabama were identified as possibly providing immigrants to Alabama, including two different populations in Georgia previously shown to be genetically isolated from each other North Georgia (NGA) and central Georgia (CGA; [22]), Mississippi (MS), Florida (FL), and Tennessee (TN, Fig 1). The Mississippi samples came from throughout the state whereas Florida samples were collected solely from the panhandle region. The samples from Tennessee came from the northwestern Great Smokey Mountains National Park. Three additional populations, North Carolina coastal (NCC), North Carolina montane (NCM) and West Virginia (WV) were also included in the study for a broader perspective on regional genetic diversity.

### DNA collection

**Fig 2:**
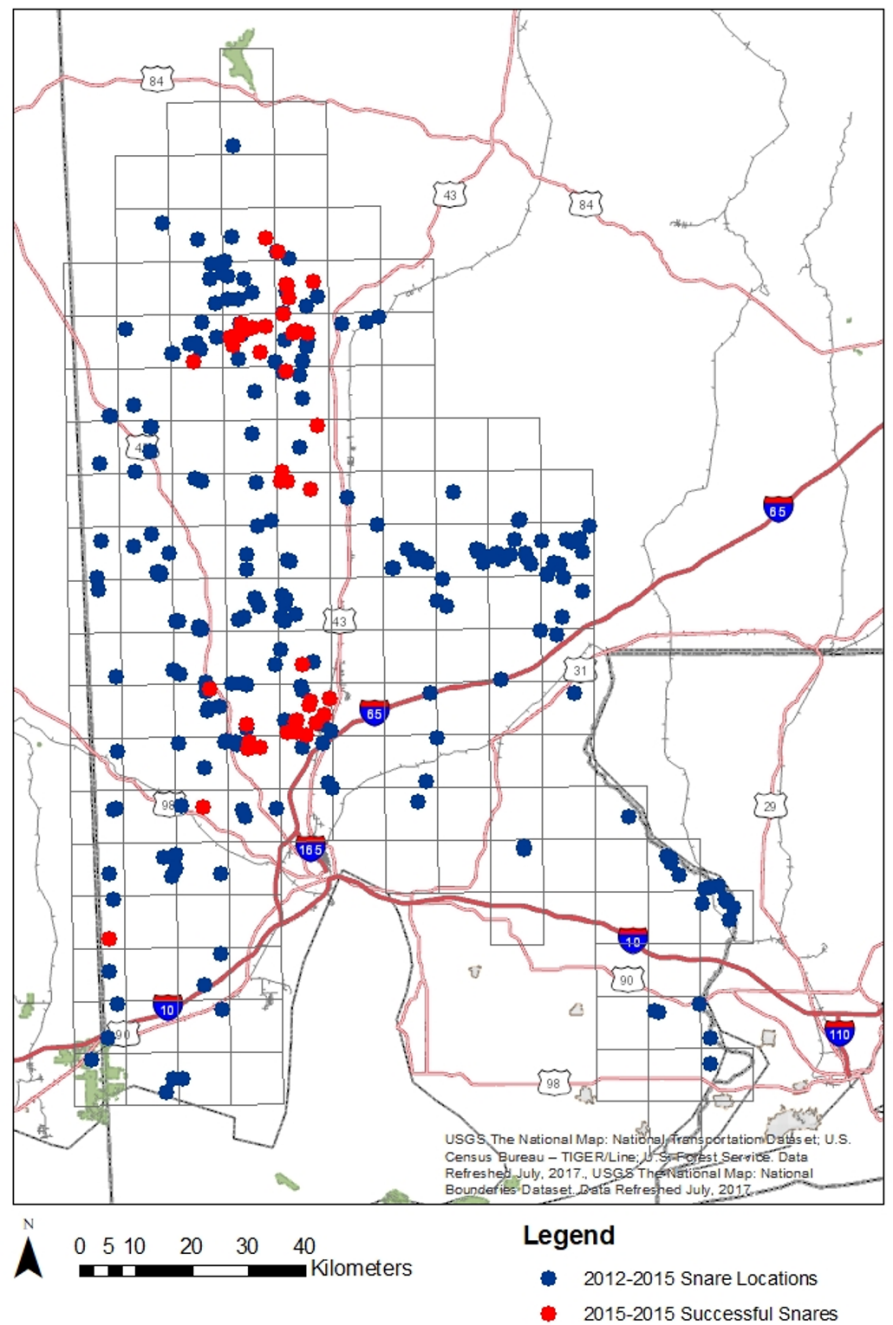
Snare placement locations for MRB. All dots represent locations where hair snares were places in the MRB, the red dots represent snares where bears were detected.

**Fig 3:**
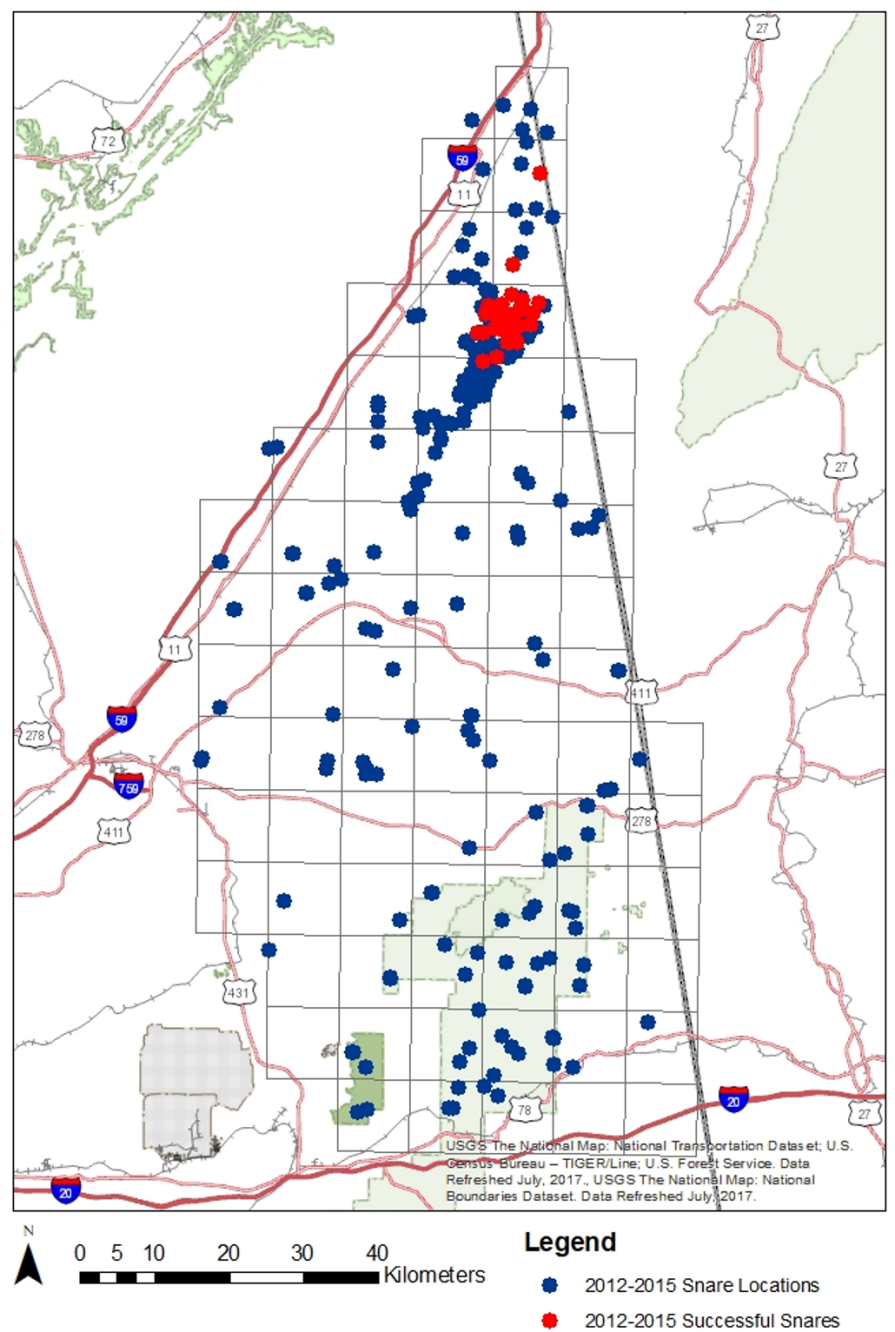
Snare placement locations for NAL. All dots represent locations where hair snares were places in the NAL, the red dots represent snares where bears were detected.

Systematic sampling for black bears took place across the known range of both Alabama populations. The study areas were overlain with a sampling grid of 64 km^2^ cells (Figs 2 and 3), which was approximately the home range size of male black bears in Alabama [19]. One hair snare was placed in each cell, with micro-site selection determined by land access, and biotic and abiotic factors affecting bear movement on the landscape (topography, water sources, food sources, etc.). In cells where bear sign (e.g. hair snare hits, camera images, tracks, scat, anecdotal reports) was found, additional hair snares were set to increase the probability of detection for females, whose home ranges are closer to 7 km^2^ [19].

Sampling was carried out from 2012–2015 by deploying minimally invasive hair snares. Hair snares consisted of a single strand of barbed wire placed around multiple trees at 45 centimeters above the ground to create a corral with a perimeter between 20–30 meters [23]. Bait was suspended in the center of the corral, such that it was greater than 2 meters from the barbed wire in any direction. Baits included canned fish and flavoring extracts applied to a tampon and suspended sufficiently high to prevent retrieval by a bear and a subsequent food reward, which could cause the animal to become trap happy or otherwise affect behavior outside of the study parameters [23].

Hair snare sampling took place from August through November when bears were in hyperphagia and most active before denning for the winter [24,25]. Sites were checked regularly with 6–8 days between checks. Hair samples were collected from the barbed wire snares using hemostats/tweezers. Collection tools were flamed both before and after collection to prevent any possible contamination or mixing of samples. Following collection of a sample, the barb it was collected from, as well as the adjacent barbs, were flamed to prevent mixing samples [23]. Collected samples were placed in paper coin envelopes and stored in a secondary container with desiccant to prevent degradation of the sample due to moisture [26].

The MRB study area was also sampled using scat-detection dogs during 2011 and 2012. Scat detection dogs allowed for more efficient and complete collection of scat along transects [27]. Dogs were trained to seek out bear scat by the EcoDogs program at Auburn University, and were each accompanied by a trained handler on all transects in addition to a biologist. Each transect was a triangle consisting of 0.5 kilometer segments, totaling a 1.5 kilometer transect. Transects were sampled across one month both fall seasons. Scats were also collected when located incidentally in both study areas throughout the study. Scrapings were taken from the most desiccated section of each scat sample to minimize potential hydrolytic degradation and were stored in 1.4ml of DETs buffer to displace any remaining water [28].

DNA samples from nearby populations were obtained using a variety of methods. Samples from central Georgia (CGA) were provided by Dr. Michael Chamberlain of the University of Georgia’s Warnell School of Forestry and Natural Resources, as tissue samples from captured individuals. Northern Georgia (NGA) samples were obtained from the Georgia Department of Natural Resources as tissue samples from hunter-harvested bears. Dr. Jerrold L. Belant of Mississippi State University provided hair samples from a hair snaring study he conducted across Mississippi (MS). The Florida (FL) samples were hair samples collected from management activities conducted by the Florida Fish and Wildlife Conservation Commission. The samples from Tennessee (TN) were obtained from Dr. Joe Clark, in the form of DNA extract from hair samples collected by Katie Settlage, for her master’s thesis research ([26,29], Fig 1). Genotypes for NCC, NCM and WB are included from previously published work by Puckett et al. (2015)[30] for a broader regional perspective of genetic diversity.

### DNA analysis

DNA analysis of all collected samples was performed at the Laboratory for Ecological, Evolutionary and Conservation Genetics in the College of Natural Resources at the University of Idaho. DNA was extracted from samples using DNeasy Blood and Tissue Kits for hair and tissue and QIAamp Fast DNA Stool Mini Kit for scat (Qiagen Inc., Valencia, CA). Hair and fecal samples were extracted in a lab space dedicated to the extraction of low quality DNA, and an extraction negative was included in each extraction to monitor for contamination. Collected scat samples were verified as bear using a mitochondrial DNA fragment species identification test as described in De Barba et al. (2014)[31]. Scat samples that were confirmed to be black bear, and all hair samples, were then identified to individual, using a microsatellite multiplex of 8 loci and a sex identification marker (G10C, G10H, G10M, G10P, G10X, G1D, Mu15, Mu23, and SE47+48; [32,33,34,35,36,37,38,39]; S1 Table). PCR products were visualized on an Applied Biosystems 3130xL genetic analyzer and allele sizes were scored and evaluated in GeneMapper 5.0 (Applied Biosystems, Foster City, California). Due to the low genetic diversity of the MRB population, an additional 6 microsatellite loci were needed to identify individuals (G10B, D1A, G10L, Mu50, G10U and G1A; [35,36,37,40,41,42]; S1 Table). Once NAL samples were assigned to an individual, a representative sample from each individual was run at the additional 6 markers to increase the accuracy of population assignment and structure tests.

The PID_sibs_ (probability that siblings share the same genotype) was calculated across all 14 microsatellite loci [43] using GenAlEx v6.5 [44]. Loci were then organized from least to most powerful PID_sibs_ value, and the product of those values calculated starting with the two least powerful and adding another locus until the product reached an acceptable threshold (S2 Table). We utilized a threshold value of 0.03, to ensure we did not identify closely related individuals as a single bear.

All samples were amplified a minimum of two times to confirm a genotype. Two amplifications were required to confirm a heterozygotic genotype for all three sample types (tissue, hair, scat). Two amplifications were sufficient for confirmation of homozygotic genotypes for tissue and hair samples, but a third consensus genotype was require for confirmation of homozygotic genotypes derived from scat samples. Genotype matching was carried out in GenAlEx v6.5 [44]. Samples were considered to be from the same individual if they were an exact match or matched at 7 of 8 loci for the NAL population and 12 of 14 for the MRB population (S2 Table) and the mismatch of the 8^th^ or 13^th^/14^th^ locus could be assumed to have been from either a potential allelic dropout or failure to amplify. To prevent overestimation of the population, genotypes that mismatched at one to three loci and where dropout or false alleles was suspected underwent additional amplifications (up to 4 total for hair and 6 total for scat) to confirm or refute differences. For NAL samples with a questionable match, the second multiplex was run, and a PID_sibs_ value of 0.03 was still maintained with only 8 loci, but out of 14 total (any mismatch of the additional loci still had to be due to potential drop out or failure to amplify). Individuals that were only represented by a single sample were required to have a Reliotype [45]value of .9 or higher and have amplified at 7 or 12 loci respectively, to be considered in any analysis, to remove potential genotyping errors.

Samples collected from comparison populations (NGA, CGA, MS, FL, TN) were run at all 14 loci. Additionally, once individuals were identified all loci and populations were evaluated as to whether they were in Hardy Weinberg Equilibrium (HWE, [46]). Linkage Equilibrium (LE) was also estimated to ensure that all measured loci were independently inherited. LE and HWE were estimated using Arlequin version 3.5.2.2 [47] and Genalex 6.5 [44] respectively. Samples (n = 5) representing the observed diversity within the Puckett library were run at the 9 shared loci (G10L, G10P, G1D, G10C, G10M, G1A, G10B, G10U and Mu23), and a correction factor was calculated for each locus, then the genotypes for each sample from the CNC, MNC and WV populations were transformed to be used for comparison.

### Population models

Once samples were identified to individual, capture histories of each individual were created for analysis in capture-mark-recapture (CMR) models. The study areas extended well beyond the observed range of bears in Alabama, which was further supported by the distribution of snare success (Figs 2 and 3). Additionally we were in close regular contact with the Alabama Department of Conservation and Natural Resources who provided us with all reports (public and Department staff) of bears outside of the study area, of which all were determined to be exploratory excursions with individuals returning to core areas within the sampling grids. The extent of the sampling study areas allowed us to assume closure within each year’s sampling period. Hair snares were not distributed in a uniform fashion on the landscape due to the hierarchical snare layout, intent on maximizing individuals sampled and the heterogeneous access to private land. To address the potential for unequal capture probability due to an uneven density of traps across the study area, a spatial covariate was calculated. The covariate was calculated as the distance between the snare where an individual was detected and the next nearest snare, which was then averaged across all detections in an individual’s capture history.

Capture histories with the calculated spatial covariate and a sex covariate were then input into the program MARK [48]. A Huggins closed capture model was used to estimate within-year population size ($\hat{N}$) for both the MRB and NAL populations [49,50]. To account for possible between year immigration/emigration, a robust design framework was applied to the NAL population’s estimates [51] and $\hat{N}$ was derived for 2012–2015. Only the 2015 MRB capture histories had a sufficient recapture rate to provide a reliable estimate of $\hat{N}$. Multiple a-priori models were tested to account for the effects of the sex and spatial covariates on capture and recapture probabilities, as well as between year variation on survival and capture and recapture for the NAL population. A likelihood ratio test (LRT) was applied to all nested pairs of models to remove more complicated models that did not account for a significant increase in explanatory power. All remaining models were then evaluated and ranked using Akaike’s Information Criterion corrected for small sample sizes (AIC_c,_[51,52,53]).

Additionally, we analyzed the data with continuous occasion CMR models in the package Capwire [54] in R studio [55,56]. The likelihood function in Capwire was developed for use with non-invasive genetic sampling studies, where individuals can be sampled multiple times during a single occasion as defined by traditional CMR models [57]. The pooling of all capture events into a continuous occasion helps for estimating small population sizes, and sparse capture histories. Within Capwire, two different assumptions about the innate capture rate of individuals were modeled. The first assumption is an even capture model (ECM) where all individuals have equal capture probabilities; and the second is a two innate rate model (TIRM), which assumes that there were two undefined groups within the sampled population that had different capture probabilities. LRTs were used to select between the ECM and TIRM models.

### Population structure

The number of populations and grouping of individuals into populations was first evaluated to remove bias associated with any *a priori* assumption of population membership of individuals. Model-based Bayesian clustering analysis was undertaken in the program STRUCTURE [58–60]. Admixture and correlated allele frequencies were assumed, and all simulations were run with 100,000 iterations of burn in and a 400,000 Markov Chain Monte Carlo run. We evaluated values of K (the number of populations modeled) from 1–12, with 10 replicates at each K to provide an averaged result. The smallest K value where the log likelihood of K begins to plateau was selected as the estimate of the actual number of populations [61] and confirmed with the Evanno method [62]. The statistics and graphs for these procedures were run in the program Structure Harvester [63]. Due to the large number of individuals identified in the MRB and its isolation, we were concerned about a potentially confounding signal from highly related family groups on the structure analysis. To reduce potential bias we ran a maximum likelihood estimate of relatedness in ML-relate [64] and selected the 30 least related individuals with which to run a second structure analysis following the same parameters as above.

Population assignment from STRUCTURE was confirmed utilizing a Discriminant Analysis of Principle Components (DAPC, [65]). DAPC uses a principle components analysis to describe as much of the within and between group variation, and the resulting principle component scores are fed into a discriminant analysis which identifies the between group genetic variation. Results were then represented graphically showing a center of commonality of principal components for each population circumscribed with an inertia ellipse which describes 95% of the variation of each population. All DAPC calculations were performed with the adegenet package [66] in RStudio [55,56].

Population structure among identified groups was evaluated utilizing Fst [67] and G”st [68,69] statistics; Fst and G”st estimates were generated using Genalex 6.5 [44].

### Genetic diversity analysis

Genetic diversity of all populations was estimated using observed and unbiased expected levels of heterozygosity (H_o_, H_e_) [70] and allelic richness, in GenAlEx v6.5 [44] and FSTAT [71], respectively. Differences among populations for all measures of diversity were assessed with a Kruskal Wallis Rank Sum test with a Nemenyi correction for multiple comparisons [72,73]. Finally the effective population size (N_e_) was calculated for both the MRB and NAL populations utilizing linkage disequilibrium methods corrected for missing data [74,75] using NeEstimator [76].

## Results

### DNA collection and analysis

A total of 1935 samples of scat and hair were collected, of which 970 yielded consensus genotypes. In the MRB study area, 135 unique individuals were identified and in the NAL 32 unique individuals were identified (S3 and S4 Tables). All of these individuals were used for genetic diversity analysis.

Samples used for population size analysis came strictly from hair snares, scat collection did not yield sufficient additional samples to justify the increased complexity of models necessary for its inclusion. Due to low recapture success in the MRB, only results from 2015 were used, which included 228 samples successfully identified to individual (S3 Table) collected from 29 of 130 deployed snares (Fig 2) and comprised of 62 unique bears. All four years of hair snare data from the NAL provided adequate capture and recapture rates for models to optimize. All four years of hair snare data yielded 874 samples, of which 427 yielded consensus genotypes which were matched to 32 unique individuals (S4 Table).

Both study areas showed a restricted distribution of detections. Though trap density, extent, and layout varied year to year in NAL, the concentration of successful snares was consistently in and around Little River Canyon National Preserve (Fig 3). The MRB population also saw a relatively limited distribution of successful snares. Despite a broad deployment of snares, bear detections were concentrated in two disjoint areas: one near Saraland in Mobile county and the other near Wagarville and Chatom in Washington county, with no detections between them (Fig 2). These two sub-populations however do not show any genetic differentiation separating them, even when the MRB was analyzed in STRUCTURE alone to allow for more minor divisions to be illuminated.

### Population models

#### MRB

The Huggins and Capwire closed capture models for the MRB provided qualitatively similar results. The two a priori Huggins models that remained after removing insignificant nested models as tested with LRT were a trap response only model and a trap response model with a constant effect from the spatial covariate (Table 1). The latter of the two models was selected by AICc, and the model estimated the population had 86.4 individuals (95% CI 64.0–165.2). Similarly, the TIRM Capwire model was selected by LRT, and estimated 86.0 individuals (95% CI 76.0–124.0).

**Table 1:** Huggins closed capture models considered for the MRB population in 2015.

Model	AICc	Delta AICc	AICc Weights	Model Likelihood	Num. Par	Deviance
{P(. t)C(.)Avmin_dis}	609.53	0.00	0.96	1.00	3.00	603.50
{P(.)C(.)}	615.72	6.19	0.04	0.05	2.00	611.71

More complex models that lacked significance in a likelihood ratio test to their reduced model pair were previously removed. P is rate of capture, C is rate of recapture, Avmin_dis is a spatial covariate that is the average minimum distance between an individual’s detection location and the next nearest available snare of all detections which was applied as a constant covariate to both P and C equally.

#### NAL

The Huggins and Capwire models for the NAL showed similar qualitative agreement, and additionally showed a general agreement in the population trend from 2012–2015. After LRT, 5 Huggins models remained for consideration, of which the clear top model accounted for variation in survival between sexes, a trap response, and an equal effect of sex on both capture and recapture probabilities (Table 2). The Huggins Robust Design model showed a clear increasing trend in $\hat{N}$ across all four years of sampling culminating in a final estimate of 24.8 bears (95% CI 22.5–36.6, Table 3). The TIRM Capwire model was selected by LRT and the population estimates support the findings of the Huggins model with a clear increasing trend across all four years, culminating in a final estimate of $\hat{N}$ of 34 bears (95% CI 26.0–43.0, Table 3).

**Table 2:** Huggins robust design model selection for the NAL population for 2012–2015.

Model	AICc	Delta AICc	AICc Weights	Model Likelihood	Num. Par	Deviance
{S(.sex)G"(.)G'(.)(P(y.)C(y.)sex)}	956.13	0.00	0.99	1.00	11.00	932.70
{S(.)G"(.)G'(.)(P(..)C(..)sex)}	966.58	10.45	0.01	0.01	5.00	956.27
{S(.sex)G"(.)G'(.)P(y.)C(y.)}	967.89	11.76	0.00	0.00	10.00	946.71
{S(.)G"(.)G'(.)P(y.)C(y.)}	970.54	14.41	0.00	0.00	10.00	949.36
{S(.sex)G"(.)G'(.)P(..)C(..)}	983.03	26.90	0.00	0.00	5.00	972.72
{S(.)G"(.)G'(.)P(..)C(..)}	985.19	29.06	0.00	0.00	5.00	974.88

S is survival between years. G" is the probability of temporary emigration in primary period i given NOT a temporary emigration at i-1, and G' is the probability of temporary emigration in primary period i given temporary emigration at i-1, for all included models both were held constant between years, but not equal to each other for a Markovian assumption of migration. P is the probability of capture and C is the probability of recapture. Y is the annual variation in either capture or recapture probability, and sex is the effect of sex on either survival, or as an equal effect on both P and C as indicated in the model name.

**Table 3:** Huggins and capwire population estimates for NAL 2012–2015.

	{S(.sex)G"(.)G'(.)(P(y.)C(y.)sex)}		95% Confidence Interval		Capwire	95% Confidence Interval	
Year	$\hat{N}$	SE	Lower	Upper	$\hat{N}$	Lower	Upper
2012	12.23	1.90	11.14	21.67	11	11	12
2013	12.86	4.41	9.06	30.18	9	8	12
2014	19.38	0.74	19.03	23.40	19	19	20
2015	24.77	2.84	22.53	36.57	34	26	43

Huggins Robust Design top model estimate of $\hat{N}$ for the NAL population for 2012–2015 (left side of table). The model assumes differential survival between sexes, but constant between years, Markovian estimates of emigration, and an equal effect of sex on both P (capture) and C (recapture). Capwire estimates of $\hat{N}$ for the NAL population for 2012–2015 (right side of the table)

### Population structure

**Fig 4:**
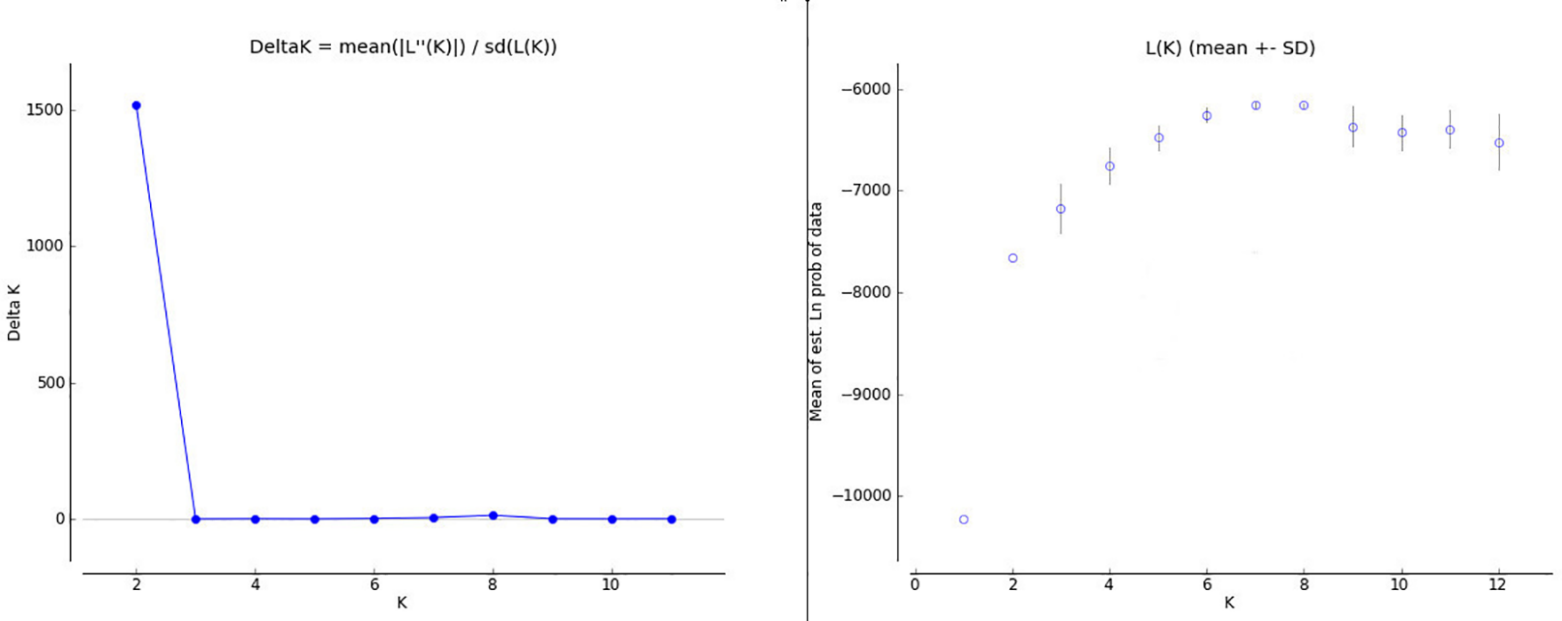
STRUCTURE K selection graphs with all samples included. Delta K (Evanno Method) for STUCTURE analysis with all samples included (left) and Log likelihood of K for STRUCTURE analysis with all samples included (right).

**Fig 5:**
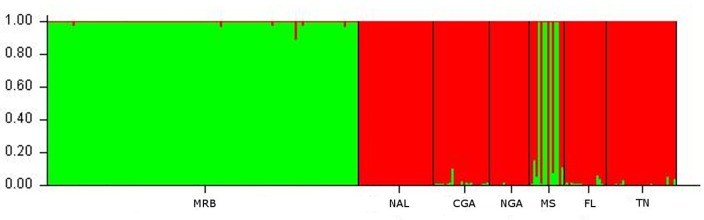
STRUCTURE bar graph with all samples included. Bar chart for K = 2 showing population assignment from STRUCTURE divided by a priori population assignment. Note the 6 individuals originally in the MS population (5), that clearly show population assignment to the MRB population.

Structure analysis was initially run on all identified individuals. A clear plateau of the L(K) value was observed after K = 6, while the Evanno method showed substantial support for selection of a K = 2 (Fig 4). The two populations assigned for K = 2, were the individuals assigned a priori to the MRB population, with the remaining populations grouped together (Fig 5). Additionally 6 bears from the MS population that were sampled close to the Alabama border were assigned to the MRB population cluster (Fig 5).

**Fig 6:**
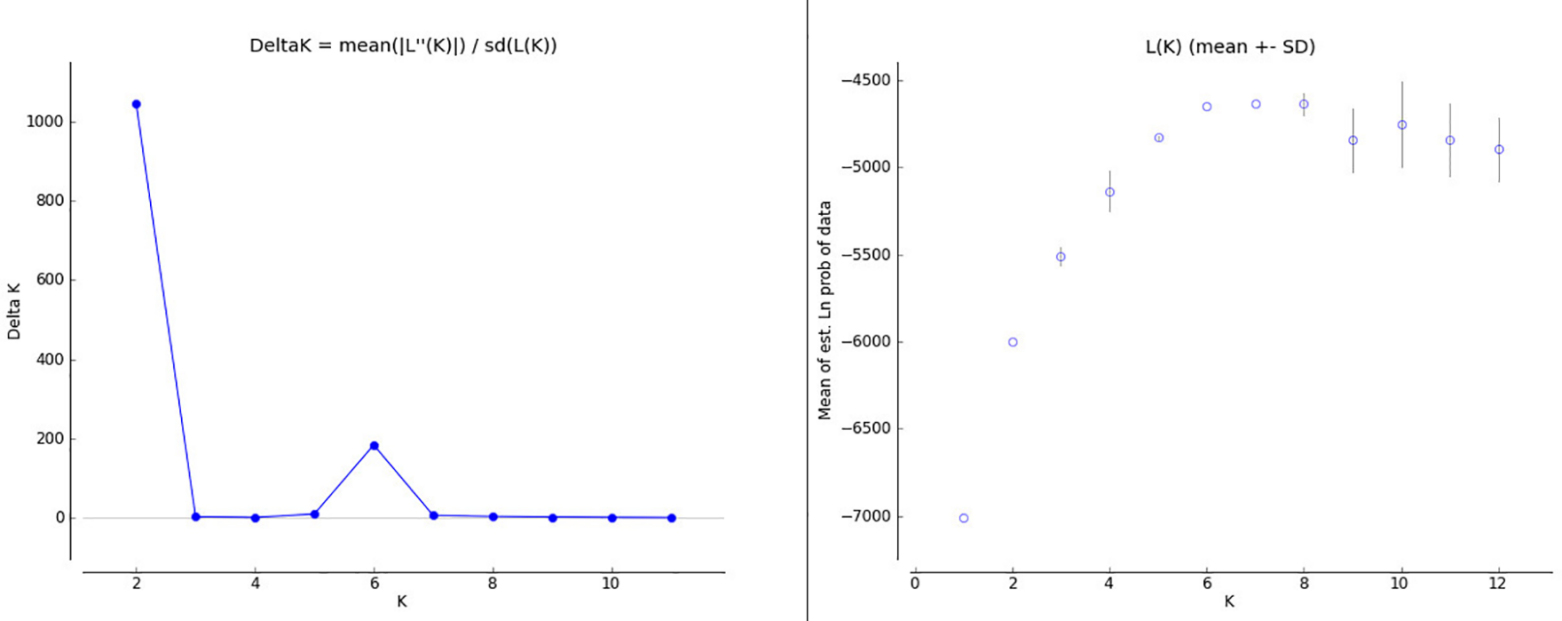
STRUCTURE K selection graphs with highly related individuals (MRB) removed. Delta K (Evanno Method) for STUCTURE analysis with highly related individuals in the MRB removed (left) and Log likelihood of K for STRUCTURE analysis with highly related individuals in the MRB removed (right).

**Fig 7:**
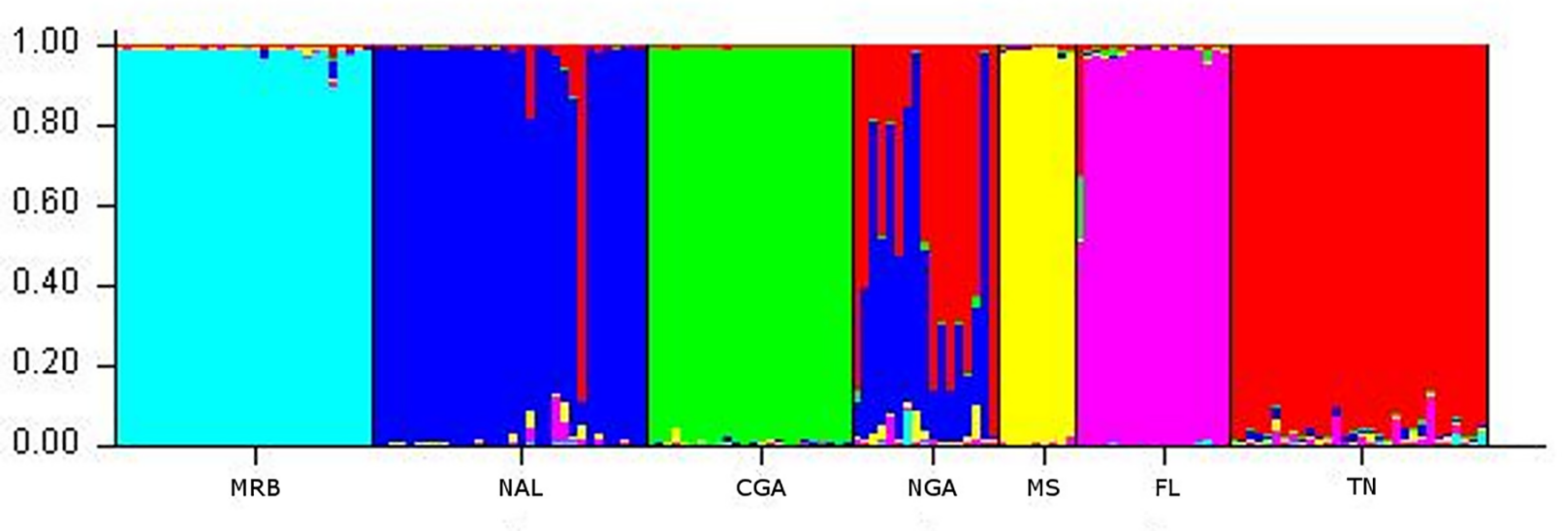
STRUCTURE bar graph with highly related individuals (MRB) removed. Bar chart showing population assignment from STUCTURE divided by a priori population assignment. The 6 individuals originally in the MS population were reassigned to the MRB for this analysis.

The second structure analysis with the MRB population reduced to 30 of the least related individuals showed a clear signal of K = 6 for both the L(K) and Evanno methods of K selection (Fig 6). K = 2 still had a higher delta K value in the Evanno graph, but the delta K at K = 6 was still substantial and in agreement with the L(K) selection. The populations were identified along their a priori population assignments except for the NGA, which was assigned as a mixture of NAL and TN populations (Fig 7).

**Fig 8:**
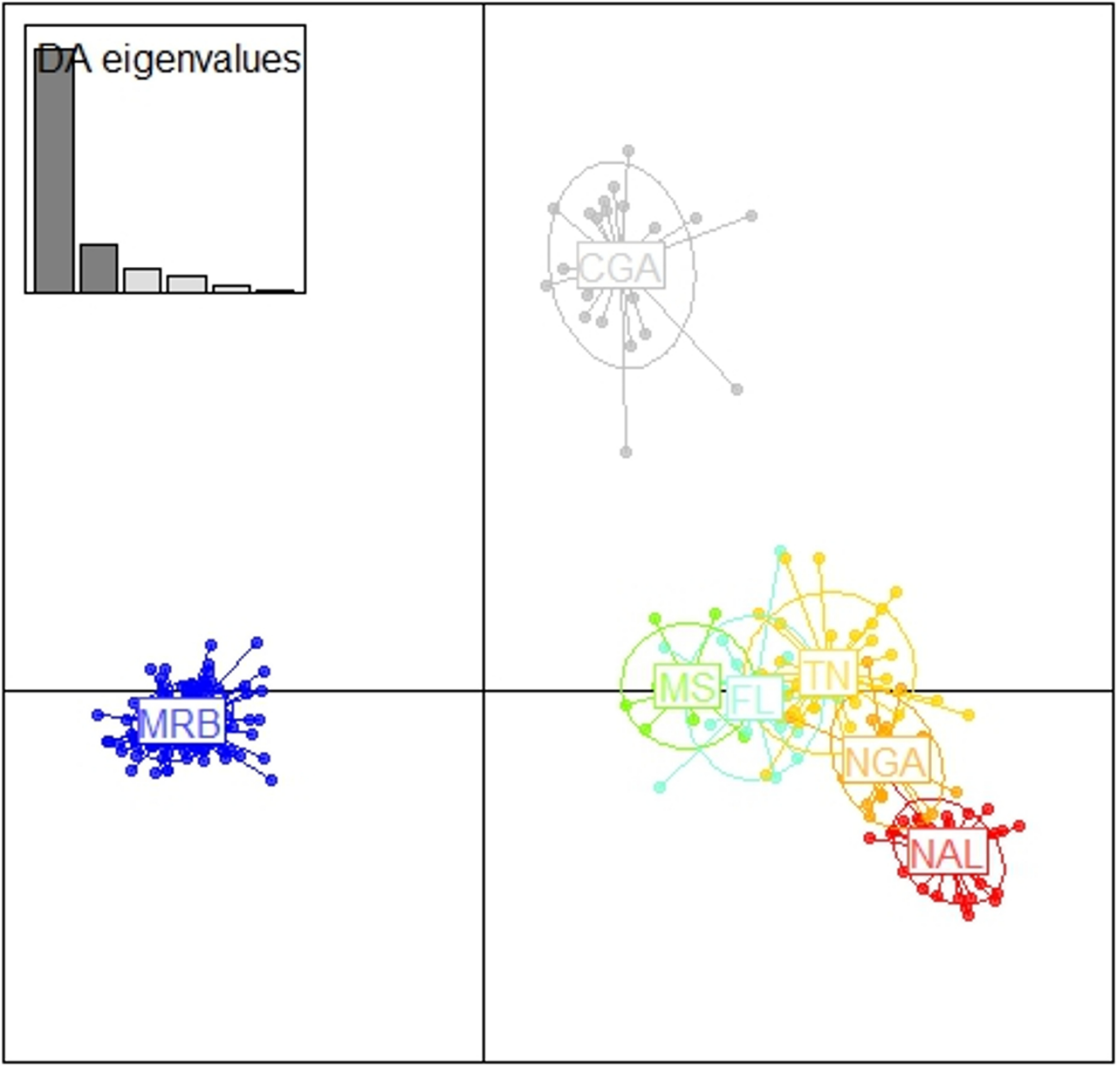
DAPC population grouping. 20 principle components and 5 discriminant functions were used. Power of the eigenvalues is shown in the upper left.

The DAPC analysis showed clear groupings of the principle components in agreement with the population assignment from STRUCTURE. To describe the data, 20 principle components were generated and 5 discriminant functions were used (Fig 8). The two populations hypothesized a priori to be isolated from surrounding populations (MRB and CGA) were shown to be distant from the core distribution of the remaining 5 populations (Fig 8). Similar to the STRUCTURE results, the NGA population sits directly between the TN and NAL populations with NGA’s 95% inertia ellipses overlapping both NAL and TN.

Both the Fst and G”st statistics show a high level of genetic structure for bears in Alabama (Table 4). The MRB population shows little to no genetic interaction with any other regional population of bears (all Fst > 0.223; all G”st > 0.763). Similarly the NAL population shows a moderate level of differentiation from all surrounding populations (Fst > 0.095, G”st >0.451) except for NGA (Fst = 0.046, G”st = 0.183). All Fst and G”st values differed from zero with a P-value of less than or equal to 0.001, based on 999 permutations.

**Table 4:** Pairwise Fst and G”st values.

	MRB	NAL	CGA	NGA	MS	FL	TN	
**MRB**		0.35	0.33	0.25	0.30	0.25	0.22	**MRB**
**NAL**	0.92		0.25	0.05	0.23	0.19	0.10	**NAL**
**CGA**	0.80	0.77		0.19	0.29	0.23	0.18	**CGA**
**NGA**	0.83	0.18	0.71		0.15	0.13	0.05	**NGA**
**MS**	0.77	0.74	0.81	0.59		0.21	0.13	**MS**
**FL**	0.76	0.74	0.78	0.63	0.74		0.10	**FL**
**TN**	0.77	0.45	0.70	0.27	0.54	0.51		**TN**
	**MRB**	**NAL**	**CGA**	**NGA**	**MS**	**FL**	**TN**	

Fst values for all populations are above the diagonal and G"st values are below the diagonal. All values have a P value of ≤0.001 based on 999 permutations.

### Genetic diversity analysis

Black bears in Alabama show low to moderate genetic diversity when compared to surrounding populations. The MRB population had the lowest allelic richness at 2.15 alleles per locus, with all comparison populations ranging from 2.43–6.00 alleles per locus (Table 5). Observed heterozygosity was also lowest in the MRB population at 0.34, with all comparison populations ranging from 0.37–0.81. Expected heterozygosity was substantially lower for the MRB than other populations at 0.33, with comparison populations ranging from 0.38–0.77. Lastly N_e_ for the MRB population was 31.0 (95% CI: 20.5–47.4). Richness and H_e_ for the MRB were significantly different for 5 of 9 pairwise population comparisons (S5 and S6 Tables) and 4 of 9 pairwise comparisons for H_o_ (S7 Table). NAL showed a more moderate level of diversity for all three measures with a richness of 3.32 alleles per locus (observed range 2.15–6.00), H_o_ of 0.56 (observed range 0.34–0.81), and H_e_ of 0.54 (observed range 0.33–0.77). Richness in the NAL population only differed significantly from the most diverse population measured (S5 Table). Finally the N_e_ for the NAL population was 3.4 (95% CI: 2.8–4.8).

**Table 5:** Allelic richness, observed heterozygosity and expected heterozygosity.

Pop	N	SE	Richness	SE	Ho	SE	He	SE
**MRB**	137.44	0.34	2.15	0.20	0.34	0.07	0.33	0.06
CGA	23.11	0.26	2.43	0.28	0.37	0.07	0.38	0.07
MS	9.00	0.00	2.89	0.20	0.36	0.07	0.42	0.05
**NAL**	31.67	0.24	3.32	0.29	0.56	0.08	0.54	0.07
FL	17.00	0.33	4.33	0.32	0.68	0.04	0.66	0.03
NGA	16.78	0.15	4.92	0.41	0.74	0.05	0.72	0.02
TN	28.44	0.71	5.03	0.31	0.67	0.04	0.71	0.04
NCC	12.78	0.57	5.53	0.45	0.79	0.03	0.74	0.02
WV	18.33	0.80	5.82	0.38	0.75	0.03	0.76	0.01
NCM	16.33	0.44	6.00	0.32	0.81	0.04	0.77	0.02

Sample populations are listed in ascending order as determined by allelic richness (Richness). N is the number of individuals contributing to each estimate, values are non integers because some genotypes were incomplete due to allelic dropout. Subsequent estimates are allelic richness (Richness), observed heterozygosity (Ho) and expected heterozygosity (He) and associated standard errors (SE).

## Discussion

With increasingly fragmented landscapes isolating wildlife into smaller populations it is important that we assess both demographic and genetic information simultaneously and with a broader regional context. Our study provides the first assessment of the genetic health of black bears in Alabama, and is the first to provide a population estimate. In addition to providing baseline population and genetic diversity estimates, we also explored regional genetic diversity for context as well as genetic connections between bear populations in the region.

Having a complete picture of black bear genetics within Alabama and how they compare and interact regionally is particularly important as our results indicated that black bear populations in Alabama were extremely small. Both the NAL and MRB populations were estimated to be less than 100 individuals, with upper confidence limits on the estimates of less than 166 individuals. While small, the NAL population showed a clear growth trend, more than doubling in 3 years (Table 3), which is promising for the continued persistence and genetic health of the population. In the MRB, however, we were unable to determine if the population was stable, growing, or declining. With only 86 individuals estimated for the core population and an N_e_ of 31, determining the trajectory of the population and breeding dynamics (i.e. dominant breeders, limited female dispersal etc.) will be critical to assessing the viability of the population.

Our spatially extensive sampling scheme allowed us to establish distribution patterns for both populations of black bears in Alabama. While previous analyses have suggested extensive suitable habitat for black bears in Alabama [77], both populations showed relatively restricted distributions. The MRB population seemed to be restricted to two pockets within its available habitat (Fig 2); however no genetic structure existed between the two, and we identified active travel corridors between them. With no observed barriers between and around these two concentrations of activity, further research is needed to explore why bears are not expanding beyond them. With the limited but increasing population seen in NAL, spatial expansion should be expected as well. Currently, the population is located in and immediately surrounding LIRI (Fig 3). However, ample habitat is available for the bear population to expand, and incidental reports of explorations by single individuals outside of the current distribution frequently are collected. Unlike the MRB, the NAL population was recently founded, and its limited distribution is more likely due to the rate of natural expansion from its core rather than an anthropogenic or biological restriction preventing it from expanding.

With the small population sizes of both populations, the genetic structure and interaction between Alabama bears and those of surrounding states is especially important for maintaining genetic diversity. The initial STRUCTURE analysis with all MRB samples included showed a clear division of the MRB population as a unique population, so much so that the Evanno method for K selection favored 2 populations the MRB and all others (Figs 4 and 5). Our results were further supported by the DAPC which showed the MRB population well separated from all other populations (Fig 8). Pairwise comparisons of Fst and G”st also show a high degree of separation between the MRB and all other populations (Table 4). The estimated level of structure indicates the population has either not interbreeding with any of its neighbors or is doing so infrequently, which could potentially lead to an inbreeding depression in the future.

Our results found that bears in eastern Mississippi were a part of the MRB population. The initial structure analysis reassigned 6 individuals from the eastern portion of the a priori MS population into the MRB population (Fig 5). Reassignment of these individuals was also supported by changes in the DAPC, Fst, and G”st values before and after STRUCTURE re-assignment. The reassignment of these individuals exemplifies the importance of defining populaton groups using genetics, rather than relying on a priori population definitions. When we removed the highly related individuals in the MRB population to eliminate any potential bias created by family groups therein, the largest amount of population variation was still described at K = 2 when evaluated by the Evanno method, seperating MRB from all other populations (Fig 6). Ultimately, however, the most appropriate value of K = 6 was selected where both delta K and L(K) agreed (Fig 6).

The NAL population of bears shows clear signs of being founded from the NGA population with a high level of separation from other populations. The second STRUCTURE analysis and DAPC clearly showed the NAL to be a distinct population, with NGA being a mixture of NAL and TN (Figs 7 and 8). These results suggest that the NGA population was founded from the TN population, but then a limited number of individuals founded the NAL population from NGA. The limited source stock for the NAL created a more unique genetic signature for the population and thus our analysis assigned NAL as unique, while NGA was assigned as between NAL and TN across all individuals. The DAPC also shows NGA as having clear overlap with both NAL and TN, but with no overlap between NAL and TN. In that analysis, all three populations also have their centers of principle components outside of the other populations 95% inertia ellipses, indicating that each population is distinct from the others. Ultimately, Fst and G”st pairwise comparisons indicate little separation between the NAL and NGA populations and only a stepwise greater structure between the NAL and TN (Table 4); these results further support a stepping stone model of migration from TN to NGA and most recently to NAL. Outside of their putative source populations, NAL shows more restricted gene flow with all other populations (Table 4).

The current low level of structure between NAL and NGA could potentially be due to the recent founding of the NAL population. Bears have been present in NAL for roughly two generations [78], which would not allow for current isolation to be detected through genetic tests which are subject to a time lag. Thus, continued genetic monitoring will be necessary to assess whether or not there is continued interaction between NAL and NGA. Continued connectivity between the NAL and NGA (or other populations) will be necessary to prevent NAL from becoming as isolated as the MRB population.

The observed low to moderate genetic diversity of black bears in Alabama is due to multiple factors. The MRB population is significantly isolated genetically from surrounding populations. This isolation, combined with its small population size and smaller N_e_, easily explains the bears’ currently low genetic diversity. Bears in NAL display a more moderate level of genetic diversity, but that diversity is still in the lower range of observed diversity of bears in the southeastern U.S. The lower diversity and small N_e_ value of NAL bears is likely due to a founder effect. However, the NAL population still appears to have some level of connection with CGA (Table 4), and the population is growing (Table 3), both of which will help to maintain the genetic diversity of the population.

This study represents the first assessment of genetic diversity and structure in Alabama black bears. Efforts should be made to continue to monitor the population size, distribution, and genetic diversity of both populations. Population size and genetic diversity are intrinsically tied to each other and monitoring both population size and genetic diversity going forward will allow managers to detect improvement or declines in population health, and respond accordingly. If barriers restricting range expansion exist for either population, further population growth will be hampered. Thus, to ensure that population growth can continue, researchers need to determine if there are any biotic or anthropogenic limitations to further range expansion for bears in Alabama. Similarly, immigration into Alabama, like the founding of the NAL population, will also be crucial. To allow for a natural infusion of new migrants to both populations, natural corridors should be identified between populations and efforts made to secure and improve them. Ideally, removing barriers to range expansion and immigration should ensure the improvement in genetic health and resulting persistence of black bears in Alabama. However, if genetic diversity continues to decline in the MRB and fitness problems are documented, translocations may need to be considered for genetic rescue and temporary infusion of novel genetic material [79]. Of course, there are numerous social, political and biological concerns with translocations, and they should only be considered as a last resort [79,80]. The results obtained in this study provide important information for management and the basis for future genetic monitoring of black bears in Alabama.

## Supporting information

S1 Table PCR protocol. Volumes of primers, and reagents (for listed concentrations) as well as μM concentration of primers used per individual sample for PCR, and thermocycler profile.
(PDF)

S2 Table PIDsib values. The PIDsib values per locus is the probability that full siblings will share a genotype at that given locus. The product of the per locus PIDsib values gives the probability that full siblings share a genotype comprised of the included loci. We set a 0.03 threshold for a genotype PIDsib value. For the NAL population the threshold was met with 7 loci with multiplex 1, but rose to 8 when including both multiplexes due to lower diversity of some of the added markers. For the MRB population the threshold was met with 12 loci.
(PDF)

S3 Table Summary of collected samples (MRB). Summary of all samples collected and successfully genotyped for individual ID in the MRB study region. Individual totals account for total unique individuals.
(PDF)

S4 Table Summary of collected samples (NAL). Summary of all sample collected and successfully genotyped in the NAL study region. Individual totals account for total unique individuals.
(PDF)

S5 Table Significance test allelic richness. Pairwise Nemenyi post hoc p-values of a Kruskal-Wallis rank sum ANOVA of allelic richness estimates. Pairwise comparisons that are below a 0.05 p-value are highlighted.
(PDF)

S6 Table Significance test H_e_. Pairwise Nemenyi post hoc p-values of a Kruskal-Wallis rank sum ANOVA of H_e_ estimates. Pairwise comparisons that are below a 0.05 p-value are highlighted.
(PDF)

S7 Table Significance test H_o._ Pairwise Nemenyi post hoc p-values of a Kruskal-Wallis rank sum ANOVA of H_o_ estimates. Pairwise comparisons that are below a 0.05 p-value are highlighted.
(PDF)
