# Supplementary material for: Genetic health and population monitoring of two small black bear (Ursus americanus) populations in Alabama, with a regional perspective of genetic diversity and exchange
Source: PLoS One. 2017 Nov 8;12(11):e0186701. doi: 10.1371/journal.pone.0186701 (PMC5695604; doi:10.1371/journal.pone.0186701)
Supplement: S1 Table — Volumes of primers, and reagents (for listed concentrations) as well as μM concentration of primers used per individual sample for PCR, and thermocycler profile. (PDF) [file pone.0186701.s001.pdf]

	MRB	NAL	CGA	NGA	MS	FL	TN	NCC	NCM	WV
MRB	-	-	-	-	-	-	-	-	-	-
NAL	0.77716	-	-	-	-	-	-	-	-	-
CGA	1	0.87096	-	-	-	-	-	-	-	-
NGA	0.01527	0.7384	0.0281	-	-	-	-	-	-	-
MS	1	0.84572	1	0.02351	-	-	-	-	-	-
FL	0.12977	0.98604	0.19942	0.99947	0.17623	-	-	-	-	-
TN	0.16716	0.99322	0.24951	0.99843	0.22246	1	-	-	-	-
NCC	0.00113	0.28866	0.00241	0.99967	0.00193	0.93475	0.90051	-	-	-
NCM	0.00088	0.25659	0.00189	0.99935	0.00151	0.91658	0.87686	1	-	-
WV	0.0088	0.63527	0.01678	1	0.01389	0.9975	0.99409	0.99996	0.99991	-
