# Supplementary material for: Genetic health and population monitoring of two small black bear (Ursus americanus) populations in Alabama, with a regional perspective of genetic diversity and exchange
Source: PLoS One. 2017 Nov 8;12(11):e0186701. doi: 10.1371/journal.pone.0186701 (PMC5695604; doi:10.1371/journal.pone.0186701)
Supplement: S2 Table — The PIDsib values per locus is the probability that full siblings will share a genotype at that given locus. The product of the per locus PIDsib values gives the probability that full siblings share a genotype comprised of the included loci. We set a 0.03 threshold for a genotype PIDsib value. For the NAL population the threshold was met with 7 loci with multiplex 1, but rose to 8 when including both multiplexes due to lower diversity of some of the added markers. For the MRB population the threshold was met with 12 loci. (PDF) [file pone.0186701.s002.pdf]

NAL PIDsib MP1			NAL PIDsib MP1 & MP2			MRB PIDsib MP1 & MP2		
**Locus**	**PIDsib**	**product**	**Locus**	**PIDsib**	**product**	**Locus**	**PIDsib**	**product**
**Mu23_1**	9.7E-01		**Mu23_1**	9.7E-01		**G10H_1**	9.9E-01	
**G10X_1**	6.7E-01	6.5E-01	**G10X_1**	6.7E-01	6.5E-01	**D1A_1**	9.9E-01	9.8E-01
**G10C_1**	5.9E-01	3.8E-01	**G10C_1**	5.9E-01	3.8E-01	**G10P_1**	9.4E-01	9.2E-01
**G1D_FIm+Rm_1**	5.7E-01	2.2E-01	**G1D_FIm+Rm_1**	5.7E-01	2.2E-01	**G1A_1**	9.1E-01	8.4E-01
**G10H_1**	5.3E-01	1.2E-01	**G1A_1**	5.5E-01	1.2E-01	**G10X_1**	8.6E-01	7.2E-01
**Mu15_1**	4.9E-01	5.8E-02	**G10H_1**	5.3E-01	6.4E-02	**G10B_1**	7.1E-01	5.1E-01
**G10P_1**	4.8E-01	2.8E-02	**D1A_1**	5.0E-01	3.2E-02	**Mu23_1**	6.8E-01	3.5E-01
**G10M_1**	4.3E-01	1.2E-02	**Mu15_1**	4.9E-01	1.6E-02	**G10L_1**	6.7E-01	2.3E-01
			**G10L_1**	4.9E-01	7.8E-03	**G10U_1**	6.5E-01	1.5E-01
			**G10P_1**	4.8E-01	3.8E-03	**G1D_FIm+Rm_1**	6.5E-01	9.8E-02
			**G10U_1**	4.8E-01	1.8E-03	**G10C_1**	5.7E-01	5.6E-02
			**G10B_1**	4.6E-01	8.3E-04	**G10M_1**	5.0E-01	2.8E-02
			**Mu50_1**	4.3E-01	3.6E-04	**Mu50_1**	5.0E-01	1.4E-02
			**G10M_1**	4.3E-01	1.6E-04	**Mu15_1**	4.9E-01	6.8E-03
