# Supplementary material for: Genetic health and population monitoring of two small black bear (Ursus americanus) populations in Alabama, with a regional perspective of genetic diversity and exchange
Source: PLoS One. 2017 Nov 8;12(11):e0186701. doi: 10.1371/journal.pone.0186701 (PMC5695604; doi:10.1371/journal.pone.0186701)
Supplement: S3 Table — Summary of all samples collected and successfully genotyped for individual ID in the MRB study region. Individual totals account for total unique individuals. (PDF) [file pone.0186701.s003.pdf]

	MRB									
	Scat			Hair			Combined			
	Collected	Genotyped	% success	Collected	Genotyped	% success	Collected	Genotyped	% success	Individuals
2011	159	24	15%	-	-	-	159	24	15%	15
2012	157	90	57%	-	-	-	157	90	57%	29
2013	-	-	-	28	15	54%	28	15	54%	10
2014	46	15	33%	218	147	67%	264	162	61%	48
2015	32	16	50%	411	230	56%	443	246	56%	61
Totals	394	145	37%	657	392	60%	1051	537	51%	133
