# Supplementary material for: Genetic health and population monitoring of two small black bear (Ursus americanus) populations in Alabama, with a regional perspective of genetic diversity and exchange
Source: PLoS One. 2017 Nov 8;12(11):e0186701. doi: 10.1371/journal.pone.0186701 (PMC5695604; doi:10.1371/journal.pone.0186701)
Supplement: S4 Table — Summary of all sample collected and successfully genotyped in the NAL study region. Individual totals account for total unique individuals. (PDF) [file pone.0186701.s004.pdf]

| | NAL | | | | | | | | | |
|---|---|---|---|---|---|---|---|---|---|---|
| | Scat | | | Hair | | | Combined | | | |
| | Collected | Genotyped | % success | Collected | Genotyped | % success | Collected | Genotyped | % success | Individuals |
| 2011 | - | - | - | - | - | | - | - | | - |
| 2012 | - | - | - | 235 | 115 | 49% | 235 | 115 | 49% | 11 |
| 2013 | - | - | - | 67 | 37 | 55% | 67 | 37 | 55% | 8 |
| 2014 | - | - | - | 407 | 181 | 44% | 407 | 181 | 44% | 19 |
| 2015 | 10 | 6 | 60% | 165 | 94 | 57% | 175 | 100 | 57% | 22 |
| Totals | 10 | 6 | 60% | 874 | 427 | 49% | 884 | 433 | 49% | 32 |
