# Supplementary material for: Genetic health and population monitoring of two small black bear (Ursus americanus) populations in Alabama, with a regional perspective of genetic diversity and exchange
Source: PLoS One. 2017 Nov 8;12(11):e0186701. doi: 10.1371/journal.pone.0186701 (PMC5695604; doi:10.1371/journal.pone.0186701)
Supplement: S5 Table — Pairwise Nemenyi post hoc p-values of a Kruskal-Wallis rank sum ANOVA of allelic richness estimates. Pairwise comparisons that are below a 0.05 p-value are highlighted. (PDF) [file pone.0186701.s005.pdf]

	MRB	NAL	CGA	NGA	MS	FL	TN	NCC	NCM	WV
MRB	-	-	-	-	-	-	-	-	-	-
NAL	0.74974	-	-	-	-	-	-	-	-	-
CGA	1	0.92249	-	-	-	-	-	-	-	-
NGA	0.00538	0.57436	0.01989	-	-	-	-	-	-	-
MS	0.97765	0.99984	0.99856	0.19542	-	-	-	-	-	-
FL	0.06638	0.9561	0.17074	0.99921	0.66663	-	-	-	-	-
TN	0.00207	0.40634	0.00852	1	0.10906	0.99322	-	-	-	-
NCC	0.00045	0.20554	0.00215	0.99988	0.03969	0.94561	1	-	-	-
NCM	1.40E-05	0.02937	8.80E-05	0.95413	0.00316	0.5679	0.98832	0.99947	-	-
WV	0.000042	0.05668	0.00024	0.98604	0.0072	0.71514	0.99779	0.99997	1	-
