# Supplementary material for: Genetic health and population monitoring of two small black bear (Ursus americanus) populations in Alabama, with a regional perspective of genetic diversity and exchange
Source: PLoS One. 2017 Nov 8;12(11):e0186701. doi: 10.1371/journal.pone.0186701 (PMC5695604; doi:10.1371/journal.pone.0186701)
Supplement: S6 Table — Pairwise Nemenyi post hoc p-values of a Kruskal-Wallis rank sum ANOVA of He estimates. Pairwise comparisons that are below a 0.05 p-value are highlighted. (PDF) [file pone.0186701.s006.pdf]

	MRB	NAL	CGA	NGA	MS	FL	TN	NCC	NCM	WV
MRB	-	-	-	-	-	-	-	-	-	-
NAL	0.89357	-	-	-	-	-	-	-	-	
CGA	1	0.98295	-	-	-	-	-	-	-	
NGA	0.01183	0.52586	0.04198	-	-	-	-	-	-	
MS	1	0.98295	1	0.04198	-	-	-	-	-	
FL	0.15672	0.96504	0.34784	0.9976	0.34784	-	-	-	-	
TN	0.00586	0.39433	0.02281	1	0.02281	0.98934	-	-	-	
NCC	0.003	0.28866	0.01262	1	0.01262	0.96972	1	-	-	
NCM	0.00024	0.07173	0.00132	0.99522	0.00132	0.7384	0.99917	0.9999	-	
WV	0.00018	0.05977	0.00101	0.99225	0.00101	0.69723	0.99843	0.99977	1	
